# Occurrence and risk factors for falls in adults with rheumatoid arthritis: a systematic review

**DOI:** 10.1186/1757-1146-6-S1-O2

**Published:** 2013-05-31

**Authors:** Angela Brenton-Rule, Keith Rome, Nicola Dalbeth

**Affiliations:** 1 Health and Rehabilitation Research Institute, AUT University, Auckland, New Zealand; 2 University of Auckland, Auckland, New Zealand

## Background

Rheumatoid arthritis (RA) is a chronic, inflammatory disease characterised by progressive joint destruction. Foot involvement is common in RA and the podiatrist is an important member of the multidisciplinary healthcare team. People with RA, of all ages, experience frequent falls and may be at greater risk of falling than the non-RA population. Falls are complex, resulting from intrinsic, behavioural and environmental risk factors. The aim of the review was to determine the occurrence and risk factors for falls in people with RA.

## Methods

A search was conducted during July and November 2012 using AMED, CINAHL, Medline, Scopus and Cochrane Library online databases. All articles were obtained from English-language peer reviewed scientific journals. To be eligible for the review a study had to include adults with diagnosed RA and have falls as a primary or secondary outcome measure.

## Results

Ten articles were identified for review. Falls incidence, over a 12 month period, ranged between 27 and 50%. Six studies evaluated potential falls risk factors. Falls were associated with decreased walking speed and standing balance, increased disease activity, co-morbid conditions, medications and fall history. Foot deformity was evaluated in only one study and found not to be associated with falls in RA.

## Conclusion

Falls in this already vulnerable group can be devastating and falls prevention is vital to the podiatric management of the RA patient. An awareness of the risk factors associated with falls in RA may help podiatrists to identify and better manage patients with increased falls risk.

